# Risk factors for loss to follow-up prior to ART initiation among patients enrolling in HIV care with CD4+ cell count ≥200 cells/μL in the multi-country MTCT-Plus Initiative

**DOI:** 10.1186/s12913-015-0898-9

**Published:** 2015-06-25

**Authors:** R Charon Gwynn, Ashraf Fawzy, Ida Viho, Yingfeng Wu, Elaine J Abrams, Denis Nash

**Affiliations:** ICAP at Columbia University, Mailman School of Public Health, New York, NY USA; Department of Medicine, Boston Medical Center, Boston, MA USA; ICAP at Columbia University, Mailman School of Public Health, Cote de Ivoire, New York, NY USA; ICAP at Columbia University, Mailman School of Public Health, New York, NY USA; ICAP at Columbia University, Mailman School of Public Health & College of Physicians & Surgeons, Columbia University, New York, NY USA; Epidemiology and Biostatistics Program, CUNY School of Public Health, New York, NY USA; Department of Health and Mental Hygiene, New York, NY USA

**Keywords:** HIV, AIDS, Anti-retroviral therapy, Pre-ART, Lost to follow up, Risk factors, Social support

## Abstract

**Background:**

In resource-limited settings, many HIV-infected patients are lost to follow-up (LTF) before starting ART; risk factors among those not eligible for ART at enrollment into care are not well described.

**Methods:**

We examined data from 4,278 adults (3,613 women, 665 men) enrolled in HIV care through March 2007 in the MTCT-Plus Initiative with a CD4 count ≥200 cells/mm^3^ and WHO stage ≤ 2 at enrollment. Patients were considered LTF if > 12 months elapsed since their last clinic visit. Gender-specific Cox regression models were used to assess LTF risk factors.

**Results:**

The proportion LTF was 8.2 % at 12 months following enrollment, and was higher among women (8.4 %) than men (7.1 %). Among women, a higher risk of LTF was associated with younger age (adjusted hazard ratio [AHR]_15–19/30+_: 2.8, 95 % CI:2.1-3.6; AHR_20–24/30+_:1.9, 95 % CI:1.7-2.2), higher baseline CD4 count (AHR_350–499/200–349_:1.5; 95 % CI:1.0-2.1; AHR_500+/200–349_:1.5; 95 % CI:1.0-2.0), and being pregnant at the last clinic visit (AHR:1.9, 95 % CI:1.4-2.5). Factors associated with a lower risk of LTF included, employment outside the home (AHR:0.73, 95 % CI:0.59-0.90), co-enrollment of a family/household member (AHR:0.40, 95 % CI:0.26-0.61), and living in a household with ≥4 people (AHR:0.74, 95 % CI:0.64-0.85). Among men, younger age (AHR_15–19/30+_: 2.1, 95 % CI:1.2-3.5 and AHR_30–34/35+_:1.5, 95 % CI:1.0-2.4) had a higher risk of LTF. Electricity in the home (AHR:0.61, 95 % CI:0.41-0.91) and living in a household with ≥4 people (AHR:0.58, 95 % CI:0.39-0.85) had a lower risk of LTF.

**Conclusions:**

Socio-economic status and social support may be important determinants of retention in patients not yet eligible for ART. Among women of child-bearing age, strategies around sustaining HIV care during and after pregnancy require attention.

## Background

Retention of patients following diagnosis and enrollment into HIV care is a prerequisite to the optimal success of HIV scale-up efforts, yet it remains a chronic challenge in both resource rich and resource poor settings [1–3]. A large proportion of patients enrolling in HIV care are in the early stages of HIV infection and not yet eligible for anti-retroviral therapy (ART) [4]. However, little is known about the magnitude and determinants of non-retention among these individuals.

A large body of research demonstrating low retention among patients that have initiated or are eligible for ART exists [4, 5]. A review of studies from Sub Saharan Africa found that only 70-77 % of patients on ART are retained at the end of two years [2]. Similarly, among patients who are eligible for ART but have not yet initiated treatment, studies also suggest high rates of non-retention, with substantial death [6, 7] and LTF [7–9]. Growing concerns about retention through all stages of the HIV care cascade, [4] not just among those identified as eligible or those initiating ART, have resulted in several recent studies that have assessed retention among patients enrolled in HIV care but who are not yet eligible for ART [10–13]. A review by Kranzer and colleagues found that retention among patients ineligible for ART ranged from 41-46 % in South Africa and was as high as 59 % in one study from Malawi [14]. Risk factors for loss to program among patients not eligible for ART differ from patients on ART, and have included higher initial CD4 count, older age, male gender, pregnancy status, longer time to clinic, and both higher and lower education levels [10–13]. While an understanding of risk factors that may impact patient retention in this critical stage in the HIV care cascade is growing, further examination of lost to follow- up and its risk factors among patients not yet eligible for ART is warranted.

We assessed the magnitude and risk factors for LTF among a population of adult men and women enrolled in HIV care prior to ART eligibility in the multi-country MTCT-Plus Initiative.

## Methods

The MTCT-Plus Initiative provided support to clinical programs in Cameroon, Cote d’Ivoire, Kenya, Mozambique, Rwanda, South Africa, Uganda, Zambia, and Thailand to implement HIV/AIDS care and treatment using a family-focused service model [15–17]. Pregnant or recently postpartum HIV-positive women identified as HIV-infected in PMTCT programs (index women) were enrolled and provided comprehensive HIV care including ART, if eligible according to the current national and/or WHO guidelines at the time of enrollment. At program entry, evaluation included a physical examination, WHO HIV disease clinical staging, and CD4+ cell count enumeration done at a local laboratory. All HIV-exposed infants and HIV-infected partners, family members, and children of index women were also eligible for enrollment into the program.

Data were collected on enrolled patients from January 8, 2003 through March 31, 2008. Patients who were enrolled on or before March 31, 2007 were considered for inclusion in this analysis. We identified 7,443 ART naïve patients older than 14 years of age (6,143 women and 1,300 men) enrolled prior to March 31, 2007 at 13 MTCT-Plus programs in 9 different countries (13 programs). We excluded two programs (Cape Town, South Africa and Mozambique) as data collection ended earlier (2007 and 2005, respectively) allowing less follow-up time. The exclusion of these sites resulted in the elimination of 627 patients (653 women and 74 men). Of the remaining 6,816 patients (5,490 women and 647 men), we restricted our analysis to patients who had at least 1 follow-up visit after an initial enrollment visit in order to assess LTF among patients who engaged in HIV care beyond the initial enrollment visit. Additionally to assess LTF risk factors for healthier HIV infected patients who would be considered ineligible for ART we limited our population to patients with a CD4 count ≥ 200 cells/mm^3^ and WHO clinical stage 1 or 2 (asymptomatic to moderately symptomatic) at enrollment. The final sample included 4,278 patients (3,613 women and 665 men).

Patients were considered LTF if their last clinic visit was at least 12 months prior to the end of data collection and they were not known to have died, withdrawn, transferred or initiated ART. Only patients who enrolled at least 12 months prior to the end of data collection were eligible to be included in the sample so that all patients had the opportunity to meet the definition for LTF. Clinic appointments were typically scheduled 3–6 months apart based on patient's clinical status with significant practice variability between clinics for which specific information was not available.

Potential risk factors for LTF were examined for women and men separately since the enrollment criteria of the MTCT-Plus Initiative targeted pregnant or post-partum women and all men who were either partners or household members of index women. Univariate analysis was conducted to identify potential risk factors for LTF. Cox regression analysis was used to estimate the relative hazards of LTF for each potential risk factor and in multivariable models. Patients who were known to have died, or who withdrew, transferred, or initiated ART were censored at the time of the event. Patients meeting the LTF definition were considered to be lost from the program 3 months after their last recorded clinic visit. A robust sandwich estimate for the covariance matrix was used to account for within site correlation [18]. Variables that were significant at P < 0.1 in the univariate analysis were tested in a multivariable model and those that remained significant with an overall P < 0.05 were retained in the final model. If variables exhibited multicolinearity, only the variable with the most significant association was retained in the model.

Enrollment year was analyzed as a categorical variable and patients who enrolled in 2007 (3.7 % of the study population) were grouped with those who enrolled in 2006. Pregnancy status and WHO stage were examined at enrollment and also as time-dependent covariates. When available, date of pregnancy provided in the clinical record was used to classify women as being pregnant at a given clinical visit, if unavailable the data was estimated based on the delivery date. Time-dependent covariates were included to assess the risk of LTF associated with the most recent measurement of pregnancy status or WHO stage. While data for CD4 tests conducted after enrollment was available, CD4 count data was not available for all patients at regular intervals therefore we did not examine CD4 as a time-dependent covariate. Among women, we estimated the attributable fraction (AF) [19] and 95 % CIs of LTF associated with pregnancy and not having adult household member enrolled in the HIV clinic using the formula AF = PE*(AHR-1)/[PE*(AHR-1) + 1] where AHR is the adjusted hazard ratio for the exposure of interest, and PE is the proportion of women with the exposure of interest.

The MTCT-Plus Initiative was approved by Columbia University’s Institutional Review Board. Data was collected for the purpose of monitoring and evaluating the service delivery program. Relevant local ethics committees reviewed and approved the program.

## Results

Table 1 shows characteristics of the study population and proportion LTF by site. The majority of patients was female (84.5 % overall). Overall 58 (1.4 %) patients were known to have died before initiating ART, 1,294 (30.2 %) initiated ART and were censored, 348 (8.1 %) were documented to have withdrawn or transferred out and 1,633 (38.2 %) never initiated ART but were alive and in care at the end of data collection. The median CD4 count at enrollment was 441 cells/mm^3^, ranging from 319 to 453 cells/mm^3^ across the 11 programs. The overall median follow-up time in the program was 1.7 years, while the proportion LTF at 12 months was 8.2 % (8.3 per 100 person-years), ranging from 0.3 % in Mulago, Uganda to 21.8 % in Kisumu Kenya. At 24 months the rate of LTF was 15.6 %, (8.4 per 100 person-years) ranging from 1.5 % in Mulago, Uganda to 35.9 % at Kisumu, Kenya. LTF at 12 or 24 months after enrollment was higher among women (8.4 % and 16.0 % respectively) compared to men (7.1 % and 13.5 % respectively). At the end of data collection 26.4 % of the total sample was LTF (26.1 % of women and 27.3 % of men) at a rate of 7.9 per 100 person-years.

**Table 1:** Site-level characteristics among patients with ≥ 1 visit after enrollment with CD4 ≥ 200 & WHO 1 or 2 at enrollment, MTCT-Plus Initiative, 2003-2008

Country/Site	Total	Females (%)	Median (IQR) FU time^1,2^	Deaths (%)	Withdrawals^3^ (%)	LTF at 12mo^2^ (%)	LTF at 24mo^2^ (%)	LTF at the end of data collection^2^ (%)
Cote d' Ivoire	588	537 (91.3)	1.7 (0.7-3)	8 (1.4)	26 (4.4)	16 (3.5)	32 (8)	43 (13)
Kisumu, Kenya	304	257 (84.5)	1.5 (0.6-2.5)	7 (2.3)	11 (3.6)	58 (21.8)	89 (35.9)	105 (50.1)
Thailand	344	262 (76.2)	1.8 (0.8-2.9)	1 (0.3)	28 (8.1)	14 (4.9)	50 (20.7)	66 (33.3)
Nsambya, Uganda	387	297 (76.7)	2.1 (1.1-3.2)	4 (1)	14 (3.6)	3 (0.9)	11 (4)	22 (11.6)
Zambia	501	443 (88.4)	1.4 (0.7-2.6)	7 (1.4)	49 (9.8)	86 (19.5)	130 (32.8)	149 (45.8)
Soweto, S.A.	496	445 (89.7)	2.7 (1–3.6)	7 (1.4)	39 (8.8)	29 (6.8)	57 (14.1)	91 (26.2)
Cato Manor, S.A.	450	406 (90.2)	1.2 (0.4-2.3)	9 (2)	140 (31.1)	33 (10)	44 (14.9)	48 (19.5)
Rwanda	191	145 (75.9)	2 (1–3.4)	1 (0.5)	13 (6.8)	16 (9.5)	28 (17.9)	44 (39)
Eldoret, Kenya	286	244 (85.3)	1.8 (0.4-3.5)	1 (0.3)	2 (0.7)	17 (7.7)	25 (12)	43 (31.2)
Mulago, Uganda	331	232 (70.1)	3.1 (1.5-3.8)	9 (2.7)	11 (3.3)	1 (0.3)	4 (1.5)	6 (4)
Cameroon	400	345 (86.3)	1.2 (0.6-1.8)	4 (1)	15 (3.8)	16 (5.3)	23 (8.6)	24 (10)
Total	4278	3613 (84.5)	1.7 (0.7-3)	58 (1.4)	348 (8.1)	289 (8.2)	493 (15.6)	641 (26.4)

^1^In years, excluding patients with no follow-up

^2^Using Kaplan-Meier product limit estimation method. Patients whose last visit was > 12 months prior to the end of data collection are considered LTF. Patients who are LTF are considered lost 90 days after their last visit

^3^Includes transfers

Table 2 shows the characteristics of the study sample. The median age for women was lower than that for men (27 vs. 33 years), and the enrollment CD4 count was higher for women than men (453 vs. 391 cells/mm^3^). At baseline 1,677 (46.4 %) women were documented as pregnant and 1,996 (55.2 %) women were pregnant at some point during the observation period. The majority of the patients (83.2 %) lived within 1 hour travel time to the clinic.

**Table 2:** Baseline characteristics of patients with ≥ 1 visit after enrollment with a CD4 count ≥ 200 & WHO 1 or 2 at enrollment, MTCT-Plus Initiative, 2003-2008

	Total	Women	Men
**Total**	4278	3613	665
**Age at enrollment (median, IQR) (years)**	28 (24–32)	27 (23–31)	33 (29–38)
15-19	198 (4.6)	195 (5.4)	3 (0.5)
20-24	1050 (24.5)	1025 (28.4)	25 (3.8)
25-29	1361 (31.8)	1218 (33.7)	143 (21.5)
30-34	1020 (23.8)	800 (22.1)	220 (33.1)
35-39	453 (10.6)	302 (8.4)	151 (22.7)
40+	196 (4.6)	73 (2)	123 (18.5)
**CD4 at enrollment (median, IQR) (cells/uL or cells/mm** ^**3**^ **)**	441 (319–608)	453 (325–620)	391 (287–522)
200-349	1352 (31.6)	1091 (30.2)	261 (39.3)
350-499	1207 (28.2)	999 (27.7)	208 (31.3)
≥500	1719 (40.2)	1523 (42.2)	196 (29.5)
**WHO Stage at enrollment**			
I	2411 (79.7)	2905 (80.4)	506 (76.1)
II	867 (20.3)	708 (19.6)	159 (23.9)
**Pregnant at enrollment**	-	1677 (46.4)	-
**≥1 Pregnancy during follow-up**	-	1996 (55.2)	-
**Disclosed HIV status**	3593 (84)	2947 (81.5)	646 (97.1)
**Marital Status**			
Legally married or living with partner	3049 (71.3)	2439 (67.5)	610 (91.7)
Not married/widowed/not living with partner	1150 (26.9)	1114 (30.8)	36 (5.4)
**Education**			
≤8 yrs	1927 (45.5)	1668 (46.2)	259 (39)
9 - 12 yrs	1789 (41.8)	1528 (42.3)	261 (39.3)
≥13 yrs	486 (11.4)	349 (9.7)	137 (20.6)
**OI Prophylaxis at enrollment**	728 (17)	607 (16.8)	121 (18.2)
**Employed outside home**	1428 (33.4)	959 (24.5)	469 (70.5)
**BMI (kg/m** ^**2**^ **)**			
<18.5	142 (3.3)	99 (2.7)	43 (6.5)
≥18.5	4120 (96.3)	3500 (96.9)	620 (93.2)
**Electricity in the home**	2636 (61.6)	2222 (61.5)	414 (62.3)
**Piped water in the home**	1976 (46.2)	1674 (46.3)	302 (45.4)
**Adult household member enrolled at clinic**	-	941 (26)	-
**Other individuals in the household (median, IQR)**	4 (3–6)	4 (3–6)	4 (3–6)
≤3	1668 (39)	1414 (39.1)	254 (38.2)
4 to 5	1315 (30.8)	1089 (30.1)	226 (34)
6 or more	1273 (29.8)	1097 (30.4)	176 (26.5)
**Number of children < 5 yo in the household (median, IQR)**	1 (0–1)	1 (0–1)	1 (0–1)
None	1165 (27.2)	985 (27.3)	180 (27.1)
1 or more	3014 (70.5)	2554 (70.7)	460 (69.2)
**Travel time to clinic**			
<1 hr	3561 (83.2)	3013 (83.4)	548 (82.4)
1-2 hrs	595 (13.9)	503 (13.9)	92 (13.8)
>2 hrs	111 (2.6)	88 (2.4)	23 (3.5)
**Year of Enrollment**			
2003	834 (19.5)	690 (19.1)	144 (21.7)
2004	1744 (40.8)	1469 (40.7)	275 (41.4)
2005	989 (23.1)	847 (23.4)	142 (21.4)
2006	554 (13.0)	474 (13.1)	80 (12)
2007	157 (3.7)	133 (3.7)	24 (3.6)

**Fig. 1:**
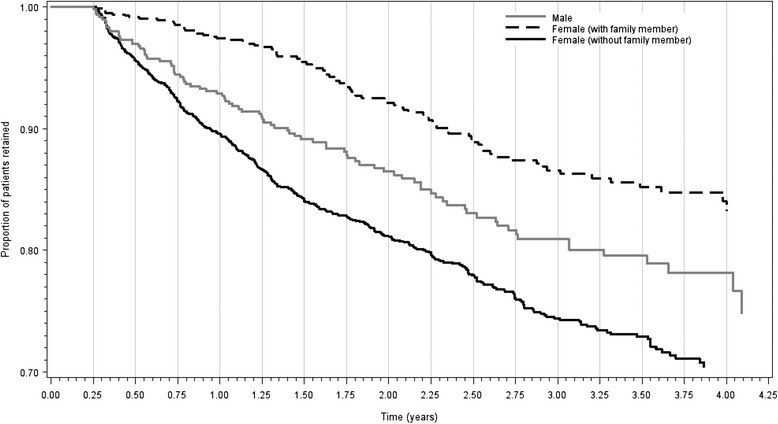
Kaplan Meier retention curve – by sex and adult household member enrolled in the program

In Kaplan-Meier curves (Fig. 1), women with another family member enrolled in the program had the lowest LTF rates over the course of follow-up (16.7 %). Higher rates were found in index women who did not have another family member enrolled (29.6 %) and men (27.3 %) who, by definition, were a husband, partner, or household member of an index woman and therefore all had another family member enrolled.

Factors examined for their association with LTF among women are shown in Table 3. Younger age was significantly associated with a higher likelihood of LTF where women aged 15–19 and 20–24 years were more likely to be LTF than women older than 30 years (adjusted hazards ratio [AHR], 2.8 95 % CI: 2.1-3.6 and 1.9, 1.7-2.2, respectively). Women with baseline CD4 count of 350–499 and ≥ 500 cells/mm^3^ were 1.5 times more likely to be LTF (95 % CI: 1.0-2.1 and 1.0-2.0 respectively) than women with a CD4 count of less than 350 cells/mm. Socio-demographic indicators that were associated with a lower likelihood of LTF included being employed outside the home, having a family member enrolled in the program, and having 4 or more people in the household (AHR: 0.73, 95 % CI: 0.59-0.90; AHR: 0.40, 0.26-0.61; and AHR: 0.74, 95 % CI: 0.64-0.85 respectively). Women who were pregnant at baseline or became pregnant during follow-up (time-dependent variable) were more likely to become LTF than women who did not become pregnant (AHR, 95 % CI: 1.9, 1.5-2.5). The amount of LTF prior to ART initiation among women attributable to pregnancy was estimated to be 33.2 % (95 % CI: 21.6 %-45.3 %), and the amount attributable to not having a family member enrolled was estimated to be 8.7 % (95 % CI: 2.8 %-15.3 %).

**Table 3:** Crude and adjusted hazard ratios for key risk factors on lost to follow-up from multivariable Cox proportional hazard model: Women

	Univariate		Multivariable	
	HR (95 % CI)	P-value	HR (95 % CI)	P-value
**Age at enrollment (years)**				
15-19	3 (2.2-4.2)	<0.0001	2.8 (2.1-3.6)	<0.0001
20-24	2.1 (1.8-2.4)	<0.0001	1.9 (1.7-2.2)	<0.0001
25-29	1.5 (1.2-1.7)	<0.0001	1.4 (1.1-1.7)	0.0010
30+	1.0	-	1.0	-
**Pregnant at baseline**	1.7 (1.2-2.4)	0.0024	-	-
**Pregnant (time dependent)**	2.1 (1.5-2.8)	<0.0001	1.9 (1.4-2.5)	<0.0001
**Baseline CD4 count (cells/mm** ^**3**^ **)**				
200-349	1.0		1.0	
350-499	1.5 (1.0-2.1)	0.0486	1.5 (1.0-2.1)	0.0439
≥500	1.5 (1.1-2.2)	0.0256	1.5 (1.0-2.0)	0.0339
**Baseline WHO stage**				
Stage I	1.0	-	-	-
Stage II	0.73 (0.44-1.2)	0.2213	-	-
**Did not disclose HIV status to partner**	1.3 (0.86-1.9)	0.2364	-	-
**Married or living with partner**	0.97 (0.83-1.1)	0.6578	-	-
**≥13 years of education**	0.59 (0.34-1.0)	0.0.497	-	-
**Receiving OI prophylaxis at enrollment**	0.68 (0.40-1.1)	0.1437	-	-
**Employed outside the home**	0.63 (0.52-0.75)	<0.0001	0.73 (0.59-0.90)	0.0038
**BMI quartiles (kg/m** ^**2**^ **)**			-	
≤21.9	1.0	-	-	-
22-24.2	1.0 (0.81-1.3)	0.8273	-	-
24.3-27.2	1.1 (0.85-1.5)	0.4089	-	-
27.3+	1.0 (0.82-1.3)	0.9097	-	-
**Electricity inside the home**	0.68 (0.49-0.93)	0.0178	-	-
**Tap water inside the home**	0.82 (0.52-1.3)	0.4046	-	-
**Adult household member enrolled in the program**	0.44 (0.28-0.67)	0.0002	0.40 (0.26-0.61)	<0.0001
**≥4 people in the household**	0.67 (0.54-0.84)	0.0004	0.74 (0.64-0.85)	<0.0001
**At least 1 child < 5 years old in the household**	0.73 (0.58-0.92)	0.0088	-	-
**>2 hours away from the clinic**	1.0 (0.68-1.5)	0.9152	-	-
**Year of enrollment**				
2003	1.0	-	-	-
2004	1.3 (1.1-1.7)	0.0087	-	-
2005	1.6 (1.1-2.2)	0.0091	-	-
2006-2007	1.6 (0.85-3.2)	0.1348	-	-

Among men (Table 4), those aged 15–29 years were twice as likely as those older than 35 to become LTF (AHR, 95 % CI: 2.1, 1.2-3.5). Having electricity in the home and living in a larger household (≥4 people) were protective against LTF (AHR, 95 % CI: 0.61, 0.41-0.91 and 0.58, 0.39-0.85 respectively). By definition, all men had another family member enrolled in the program (the index woman).

**Table 4:** Crude and adjusted hazard ratios for key risk factors on lost to follow-up from multivariable Cox proportional hazard model: Men

	Univariate		Multivariable	
	HR (95 % CI)	P-value	HR (95 % CI)	P-value
**Age at enrollment**				
15-29	2.4 (1.3-4.4)	0.0050	2.1 (1.2-3.5)	0.0073
30-34	1.5 (1.0-2.3)	0.0335	1.5 (1.0-2.4)	0.0468
35+	1.0	-	-	-
**Baseline CD4 count (cells/mm** ^**3**^ **)**				
200-349	1.0			
350-499	1.2 (0.72-2.0)	0.4971	-	-
≥500	1.7 (1.1-2.7)	0.0227	-	-
**Baseline WHO stage**				
Stage I	1.0		-	-
Stage II	0.59 (0.26-1.3)	0.1996	-	-
**Did not disclose HIV status to partner**	1.9 (0.59-6.4)	0.2721	-	-
**Married or living with partner**	0.99 (0.41-2.4)	0.9739	-	-
**≥13 years of education**	0.53 (0.34-0.84)	0.0069	-	-
**Receiving OI prophylaxis at enrollment**	0.81 (0.43-1.5)	0.5056	-	-
**Employed outside the home**	0.66 (0.36-1.2)	0.1735	-	-
**BMI quartiles**			-	
<20.2	1.0	-	-	-
20.3-21.9	1.0 (0.71-1.5)	0.8412	-	-
22-24.2	0.58 (0.32-1.1)	0.0744	-	-
24.3+	0.76 (0.45-1.3)	0.3201	-	-
**Electricity inside the home**	0.65 (0.39-1.1)	0.0883	0.61 (0.41-0.91)	0.0158
**Tap water inside the home**	1.0 (0.51-2.0)	0.9792	-	-
**≥4 people in the household**	0.48 (0.32-0.71)	0.0002	0.58 (0.39-0.85)	0.0053
**At least 1 child < 5 years old in the household**	0.77 (0.51-1.2)	0.2151	-	-
**>2 hours away from the clinic**	1.3 (0.51-3.1)	0.6191	-	-
**Year of enrollment**				
2003	1.0	-	-	-
2004	1.0 (0.57-1.8)	0.9943	-	-
2005	1.3 (0.74-2.1)	0.3940	-	-
2006-2007	1.1 (0.41-2.7)	0.8960	-	-

## Discussion

We assessed risk factors for LTF among HIV-infected, adults enrolled in HIV-care within the MTCT-Plus Initiative who were not ART-eligible at enrollment. LTF rates in our sample at 12 months after enrollment were relatively low (8.2 %) compared with other studies in Sub-saharan Africa [13, 14]. Among both men and women, we found that younger age was a risk factor for LTF. Women with higher CD4 count and those who were pregnant were more likely to be lost to follow-up, while having another adult household member enrolled in the program (a possible proxy for social support) was protective against LTF. These findings contribute to the growing body of research on outcomes among adults enrolled in HIV care who are not yet eligible for ART [10–13].

While the population of patients in our study did not meet ART eligibility criteria at the time the study was conducted, most would be eligible under current WHO recommendations which raise the CD4 threshold for ART initiation to 500 [20, 21]. While the implementation of such guidelines in resource-limited setting may be challenged by demand and funding constraints, a significantly increased number of healthier patients will potentially need to adhere to ART medications and routine clinical visits. These findings suggest that less immunocompromised, healthier patients remain at an elevated risk for LTF from HIV care. The logic that healthier patients are less likely to remain in care may also hold important implications for patients enrolled in ART at higher CD4 levels.

The higher retention rate among men than among women has generally not been observed in other studies of retention among pre ART patients in sub-Saharan Africa [12, 13]. This is likely a result of the nature of the MTCT-Plus program, which is a family-focused model of care that recruits women and their family/household members, including male partners. Therefore, by design, any male in the program has at least one other female household member enrolled with them. As a result of this program model, risk factors for men enrolled in the MTCT-Plus program may be different from those of men enrolling in care independent of a partner. Mutual support may come from disclosure of HIV status, and may be relevant for strategies aimed at improving retention in HIV care, including among men in sub-Saharan Africa, who generally start treatment later [22] and have worse retention in HIV care [23, 24].

The finding that women who were pregnant at enrollment or subsequent visits were at higher risk for LTF than non-pregnant women is especially concerning. In our study population about 55.2 % of women were either pregnant at enrollment or at some point during follow-up. Based on these findings we estimated that up to 33.2 % of all LTF in our study population may be attributable to pregnancy in women. Our findings are consistent with at least one other recent study that Mozambican women who were pregnant at enrollment were 1.3 times more likely to be lost to follow-up than those who were not pregnant, with LTF tending to occur at or near 39 weeks gestation [13]. Similar findings have been observed among pregnant women in South Africa who were more likely to be LTF both prior to and following ART initiation [25]. Possible reasons may include challenges of securing childcare to attend follow-up clinic visits, undocumented transfer to other HIV care delivery points (such as the ANC, PMTCT, or other care and treatment sites). Given the likelihood that many women will become pregnant at some point during their lifelong HIV care, and the potential for prevention of HIV infection in newborns, HIV programs should strive to strengthen screening, counseling, sustained outreach and linkages to and retention in care with immediate ART initiation. As more countries move toward immediate initiation on ART for all HIV positive pregnant women through Option B+ ^24^, LTF among pregnant women in HIV care will need to be addressed.

Our findings are consistent with qualitative studies that have found that social support likely influences retention in care following ART initiation [26–28]. We found that women with a household member enrolled in the program had the highest retention rates while women with no adult household member enrolled had the lowest (Fig. 1). Since all men in our study were enrolled with a family member (the index women) the impact of partner support on retention in men could not be directly assessed in our sample. Not having another adult household member enrolled explained up to 8.7 % of LTF among women in our sample. We also found that both men and women living in larger households are more likely to stay in care, which may also be a reflection of the influence of social support on retention. Contrary to this finding, Lessells and colleagues found that women from households with 10 or more persons were less likely to be retained in care, suggesting that commitment and possibly financial constraints resulting from family size may result in attrition from care [12]. Our finding that larger families enhance retention in care may have resulted from the unique model of care of the MTCTplus program. Social support systems are an integral part of managing patients care [29], and such systems can be especially helpful for pre-ART care where the patient may not yet be experiencing HIV-related symptoms.

We also found that being employed outside the home in women and having electricity in the home in men was protective against LTF. Electricity and employment are suggestive of higher socio-economic status, and the resulting association indicates that there may be economic barriers to receiving HIV care prior to eligibility and start of ART. Even when HIV care services are free, social or economic hardship can impact retention. Ochieng-Ooko et al. found that socioeconomic challenges, such as work and family commitments as well as transportation costs were important reasons for missing clinic appointments [30]. Other studies have found that food insecurity is a risk factor for poor retention [31, 32], Helping patients overcome economic or financial obstacles is an ongoing challenge for programs aiming to improve retention, especially in pre-ART care prior to ART eligibility, where clinic appointments are less frequent and many patients may feel quite healthy.

There are important limitations that may impact interpretation of our findings. First, it is possible that some patients considered LTF may have enrolled in care at another facility or service delivery point within the same health facility (e.g., PMTCT through the ANC) during study follow-up, but were not properly documented as transfers (silent transfers). Given the rapid scale up of HIV programs in resource limited settings in recent years, it is possible that persons living further from the clinic might seek care at a more conveniently located facility instead of the original MTCT-Plus site. As a consequence we would expect LTF to increase with travel time to clinic and year of enrollment, as more clinical services became readily available during the later years of the study period. However our data did not reveal an association between travel time and LTF or a consistent trend with year of enrollment, as has been seen with ART patients in large scale-up programs [33].

Similarly, some patients classified as LTF may represent unascertained deaths, potentially introducing bias into the association between the risk factor and LTF. This type of misclassification may also play a role in the finding that pregnant women are more likely to be lost to follow-up, as maternal mortality is substantial in sub-Saharan Africa where 1 in 31 women die during childbirth (ranging from 470–930 deaths per 100,000 live births) [34]. Geng et al. suggest that true causal relationship can be assessed through ascertainment of patients status who are lost to follow-up, using a sampling based approach and adjustment of estimates [24]. However, while misclassification of HIV-related deaths as LTF is a possibility, this may be less of a concern in our study population, which was limited to those with CD4 ≥ 200 and WHO stage I or II at baseline. Additionally, our definition of LTF may not accurately categorize patients who leave care for a prolonged period but then return to care and should be considered LTF. This misclassification is likely minimal as the median time between visits was 56 days, and only 237 (5 %) of patients who were considered to be retained in care had 12 month or longer lapses between prior clinic visits, suggesting that most patients with lengthy lapses are in fact LTF.

Another important limitation is the generalizability of the findings to general HIV care populations. Specifically, males in the study were closely related to an index women already enrolled in the program (either as a husband, partner or household member) and are not representative of the general population as they all have had the index woman’s HIV status disclosed to them and have subsequently agreed to HIV testing and enrollment into HIV care. Additionally, the focus of this study is patients who have initially engaged in HIV care, by attending at least one visit, and generalization to the entire HIV-infected population accessing care should be cautioned.

Finally, we observed variations in LTF by site (Table 1). Standardized protocols developed for the MTCT-Plus Study were implemented across the study facilities; however there may have been some differences in the way that facilities implemented the protocols. Such differences were not systematically documented, and could not be accounted for in or explained by this analysis.

## Conclusions

Retention in HIV care prior to ART initiation is a pre-requisite to: 1) optimal prophylaxis, diagnosis, and treatment of opportunistic illnesses; 2) effective secondary prevention of HIV transmission; and 3) more timely ART initiation. This study provides insights into risk factors for LTF among patients enrolled in care with CD4 ≥ 200 cells/μL and WHO stage ≤ 2 at enrollment who were not yet on ART in the family-focused MTCT-Plus Initiative. *S*ocio-demographic and social support factors were protective against LTF in both men and women; our findings suggest that both groups may benefit substantially from enrolling with a family or other household member that they feel comfortable enough to disclose to. Overcoming broad barriers to care associated with lower SES and lack of social support will likely remain a challenge to care and treatment programs. However gender-specific risk factors also compromise program retention, of particular importance is the risk of LTF among pregnant women enrolled in HIV care. Among women of child-bearing age, counseling and strategies around sustaining continuity of HIV care during and after the pregnancy and engagement in ART and PMTCT are particularly important. Retaining healthier patients in care and ART is critical to minimizing HIV morbidity and reducing HIV transmission, effective strategies must be developed that are customized to the needs of the patient population.
